# Broad Spectrum Algae Compounds Against Viruses

**DOI:** 10.3389/fmicb.2021.809296

**Published:** 2022-01-14

**Authors:** Jacqueline Graff Reis, Rafael Dorighello Cadamuro, Ariadne Cristiane Cabral, Izabella Thaís da Silva, David Rodríguez-Lázaro, Gislaine Fongaro

**Affiliations:** ^1^Laboratory of Applied Virology, Department of Microbiology, Immunology, and Parasitology, Federal University of Santa Catarina, Florianópolis, Brazil; ^2^Department of Dentistry, Federal University of Santa Catarina, Florianópolis, Brazil; ^3^Department of Pharmaceutical Sciences, Federal University of Santa Catarina, Florianópolis, Brazil; ^4^Microbiology Division, Faculty of Sciences, University of Burgos, Burgos, Spain; ^5^Research Centre for Emerging Pathogens and Global Health, University of Burgos, Burgos, Spain

**Keywords:** virucidal, algae, antiviral, health, mechanisms

## Abstract

The pharmaceutical industry is currently trying to develop new bioactive compounds to inactivate both enveloped and non-enveloped viruses for therapeutic purposes. Consequently, microalgal and macroalgal bioactive compounds are being explored by pharmaceutical, as well as biotechnology and food industries. In this review, we show how compounds produced by algae include important candidates for viral control applications. We discuss their mechanisms of action and activity against enveloped and non-enveloped viruses, including those causing infections by enteric, parenteral, and respiratory routes. Indeed, algal products have potential in human and animal medicine.

## Introduction

Many significant zoonotic pathogens are viruses and they have substantial infection-related impacts on public health worldwide. Non-enveloped enteric viruses are persistent in the environment (in water, soil and sewage, and on surfaces), resisting pH ranges of up to 3 to 9, temperature variations, and radiation (Evans, 1982; Taylor et al., 2001; Olival et al., 2017). Such viruses are a common cause of foodborne contamination (Carter, 2005; Elizondo-Gonzalez et al., 2012), which is responsible for 1.1 million hospitalizations and 218,000 deaths/year of children in countries with poor sanitary conditions (Patel et al., 2008). Enveloped viruses, with their environmental dissemination by air and aerosols, are responsible for pandemic events such as those involving Influenza and Coronavirus (Medina and García-Sastre, 2011; Chiu et al., 2012; Wang et al., 2018; Glass et al., 2020). Some enveloped viruses demonstrated high capability to cause epidemic episodes, such as Dengue virus on 2019 infecting 6,162,394 and causing death of 3,930 people. Ebola virus on Uganda/Congo region between 2018/2020, causing 3,453 infections and 2,273 deaths, other viruses as Nipah, Yellow fever and Zika caused epidemics through the worldwide (Agca et al., 2021; Anand et al., 2021; Yoon et al., 2021).

Enteric and respiratory viruses present enormous challenges both for human and animal medicine, and there is therefore a need for new antiviral solutions for fighting viruses with their worldwide economic and social consequences. Recent respiratory viral epidemics such as the worldwide outbreaks of swine flu, avian flu, and coronavirus has increased interest in the development of antiviral drugs (Prasse et al., 2010; Feng et al., 2018; Ye et al., 2020). Exploring natural compounds is an important approach to obtaining new virucides and antivirals. Macroalgal marine polysaccharides are potential candidates for human and animal medicine and have attracted the attention of the scientific community for applications in biotechnology (Dias et al., 2012; Wang et al., 2012).

Macroalgae are a phylogenetically artificial group of multicellular, macroscopic, eukaryotic, photoautotrophic organisms, mostly benthic (many being commonly known as seaweed), which are classified into three large groups: Chlorophyta (green algae), Rhodophyta (red algae), and Phaeophyceae (brown algae) (Leandro et al., 2019).

The components of macroalgae vary from simple to complex compounds, including polysaccharides (e.g., fucoidan, alginate, laminarin, carrageenan), phenolics and phlorotannins (e.g., flavonoids, lignans, tannins), protein and peptides (themselves made up of amino acids including leucine, glutamic acid, tryptophan), lipids, terpenoids and steroids (e.g., carotenoids), vitamins and minerals (Spolaore et al., 2006; Onofrejová et al., 2010; Balboa et al., 2013). Freshwater and marine microalgae contain compounds of high relevance to health, for example vitamins, proteins with essential amino acids, fatty acids, polysaccharides, minerals, enzymes, fibers and photosynthetic pigments such as carotenoids and chlorophylls (Montalvão et al., 2016; Silva et al., 2018). Various bioactive compounds from algae are attracting growing interest because of their antitumor, antiviral, anticoagulant, and antioxidant properties (Wang et al., 2012; Wang et al., 2018).

Algae have a variety of broad-spectra activities against viruses and low cytotoxicity, both *in vitro* and *in vivo*. However, their potential is still underexplored by the pharmaceutical industry, and only 9% of medicines of natural origin come from algae (Kumar Jha and Zi-Rong, 2004). Besides that, there are many *in vitro* studies proving the antiviral effects of algae, there have been few studies of their efficiency *in vivo* and in the environment. Indeed, there is an urgent need for further investigations of this type (Rosa et al., 2020).

This review presents selected compounds from algae as candidates for the control of viruses with applications in the phytochemical, pharmaceutical, and sanitizing sectors.

## Obtaining Crude and Fractionated Extracts for Antiviral and Virucidal Assays From Algae

Bioactive compounds can be extracted from macroalgae or microalgae using various methods. The most common for extracting bioactive compounds from marine samples is solid-liquid extraction (SLE), involving solvents to extract soluble constituents from a solid or semisolid matrix. The downside of this method is its long extraction time and high solvent consumption (Wang et al., 2018; Jacobsen et al., 2019). Other relevant techniques are solid-phase extraction (SPE), supercritical fluid extraction (SFE), ultrasound-assisted extraction (UAE), microwave-assisted extraction (MAE), and pressurized liquid extraction (PLE); SFE, UAE, and MAE are the most commonly used for macroalgae (Kaufmann and Christen, 2002; Ciko et al., 2018).

Obtention of compounds of interest from marine macroalgae and microalgae involves the following major steps: (1) collection and taxonomic identification of samples; (2) drying; (3) extraction through the use of solvent; (4) filtration and concentration by evaporation (Fayzunnessa et al., 2011; El-Baz et al., 2013; Choi et al., 2014).

Cytotoxic, antivirus, and virucide assays and other cell culture techniques are used to screen candidates. Cytotoxic assays identify compounds that are toxic to healthy cells. A typical protocol is to cultivate an appropriate cell line in an Eagle medium at 30° with 5% of CO_2_ in a layer in a 96-well plate, and applying serial dilutions of compounds of interest. After 7 days of incubation, viable cells are revealed by using chemicals such as sulphorhodamine B and tetrazolium dye 3-(4,5-dimethylthiazol-2-yl)-2,5-diphenyltetrazolium bromide (MTT) (Chacon et al., 1996; Vichai and Kirtikara, 2006).

Antiviral assays identify compounds that for example inhibit viral replication. Such assays generally involve placing viruses in contact with permissive cells and allowing the cycle of replication to begin. The antiviral compound being tested is then applied and successfully replicated viruses counted as plaque-formed units (PFU) after revelation by staining cells with crystal violet. Percentages of viral inhibition and of cells free of infection can then be calculated.

Virucidal assays identify compounds that kill viruses, by for example, inactivating recognition proteins. Viruses are exposed to the sample under study and then a known quantity of virus is used to inoculate cells in 6-well plates; in some protocols, agarose is use to stabilize neighboring cells to favor the transmission of viruses. Viral neutralization is quantified by counting PFU after viable cells are colored with crystal violet (Jothikumar et al., 2005). This assay is important for evaluating compounds that may be able to inactivate viruses in the environment (Victoria et al., 2009; Rigotto et al., 2011; Célia da Silva Lanna et al., 2019).

## Discussion

Algae and their extracts have numerous applications and have historically stimulated significant economic interest, mainly as a source of new drugs such as antivirals. therefore, we report crude extracts and compounds isolated from algae that showed antiviral activity against both enveloped and non-enveloped viruses. Some examples of these compounds and extracts from algae with antiviral activity are listed in Table 1.

**TABLE 1:** Species and compounds from algae used as antivirals.

Species	Compounds	Applications	References
**Macroalgae**			
–	Iota-carrageenan	HRV1A, HRV2, HRV8, HRV14, HRV16, HRV83, HRV84	Grassauer et al., 2008
*Stypopodium zonale*	Meroditerpene epitaondiol	HMPV	
*Laminaria japonica*	Sulphated polysaccharide fucoidan	H5N1, RSV	Cao et al., 2016
*Chondrus armatus* *Laminaria cichorioides, Laminaria japonica*	ι-carrageenans, fucoidan	Hantavirus	Pavliga et al., 2016
*Griffithsia sp*.	Protein griffithsin	SARS-CoV (Urbani strain), HIV, HCV, HSV-2, JEV, PEDV	Mori et al., 2005, O’Keefe et al., 2009, O’Keefe et al., 2010, Meuleman et al., 2011, Ishag et al., 2013, Takebe et al., 2013, Levendosky et al., 2015
*Griffithsia sp.*	Grifonin-1	HIV-1	Micewicz et al., 2010
*Laurencia obtusa*	Polysaccharide	HCV	Gheda et al., 2016
*Cladosiphon okamuranus*	Fucoidan	DENV-2	Hidari et al., 2008
–	λ-carrageenans, ι-carrageenans	DENV-2, DENV-3	Talarico and Damonte, 2007
–	Fucoidan	NDV	Elizondo-Gonzalez et al., 2012
*Cladosiphon okamuranus*	Fucoidan	CDV	Trejo-Avila et al., 2014
*Eisenia bicyclis*	Dieckol/phlorofucofuroeckol-A	FCV, murine norovirus	Choi et al., 2014, Eom et al., 2015
*Nicotiana benthamiana*	Griffithsin/Carrageenan	HSV-2/human papillomavirus	Levendosky et al., 2015
*Schizymenia binderi*	Sulphated galactan	HSV-1, HSV-2	Matsuhiro et al., 2005
*Gracilaria corticata*	Sulphated galactan	HSV-1, HSV-2	Mazumder et al., 2002
*Padina pavonica, Sargassum vulgare, Pterocladia capillace, Laurencia obtusa*	Sulphated polysaccharides	HCV	Gheda et al., 2016
**Microalgae**			
*Chlorella vulgaris*	Polysaccharide	HSV-1	Santoyo et al., 2010
*Gyrodinium impudium*	Sulphated polysaccharide p-KG03	IAV, EMCV	Kim et al., 2012
*Spirulina platensis*	Sulphoquinovosyl diacylglycerol	Adenovirus 40-7, Coxsackievirus B4, Astrovirus type 1, Rotavirus Wa, HSV-1	Abdo et al., 2012; El-Baz et al., 2013
*Cochlodinium polykrikoides*	Sulphated polysaccharides A1 and A2	Influenza A and B viruses, RSV-A, RSV-B	Hasui et al., 1995
*Spirulina platensis*	Calcium spirulan	HIV1, HIV2, HSV1, HSV2, HCMV, MuV, IAV	Hayashi et al., 1996
*Porphyridium cruentum*	–	HH3, VV, ASFV, VHSV	Fabregas et al., 1999
*Chlorella autotrophica*	–	VHSV, ASFV	Fabregas et al., 1999

### Crude Extracts From Algae Against Viruses

Red seaweed *Osmundaria obtusiloba* has been reported to have higher antiviral and virucidal effects against the Chikungunya virus (CHIKV) than ribavirin, which is used as a drug to control the virus (Cirne-Santos et al., 2019). The λ- and ι-carrageenans obtained from *Osmundaria obtusiloba* have potent antiviral activity against dengue virus type 2 (DENV-2) and type 3 (DENV-3) (Talarico and Damonte, 2007).

An ethanol extract from the microalgae *Spirulina platensis* has antiviral effects against Adenovirus type 40, a non-enveloped virus (Abdo et al., 2012). This virus causes gastroenteritis and mortality especially in children. *S. platensis* also has antiviral effects against Adenovirus type 7, Astrovirus type 1, Coxsackievirus B4 and Rotavirus Wa strain (El-Baz et al., 2013), all of which cause gastroenteritis in humans.

*Gyrodinium impudium* is a marine microalga with antiviral effects against encephalomyocarditis (EMCV) non-enveloped viruses. EMCV infection causes death among pigs in production units, primates in research centers, and animals in zoos (Kim et al., 2012).

Extracts of some algae have been fed to some types of shrimp to reduce the impact of the white spot syndrome virus (WSSV); this macroalgae and microalgae diet appears to have improved innate immunity and increased the resistance of shrimp to infection by WSSV (Chotigeat et al., 2004; Immanuel et al., 2012; Sivagnanavelmurugan et al., 2012; Charoonnart et al., 2019). Seaweed extracts have also been used in fish diets and have shown promising antiviral effects against the salmon anaemia virus (ISA) and the enveloped RNA virus (Lozano et al., 2016).

### Biocompounds Isolated From Algae Against Viruses

A wide variety of compounds obtained of micro and macroalgae has already been explored and tested against viruses and their cell infection capabilities.

Several studies of macroalgae compounds have shown promising antiviral effects against viruses that cause animal diseases causing serious economic losses. There are no effective treatments, either antivirals or vaccines, currently available against many of these diseases. A study in 2012 reported that the brown algae compound fucoid had an antiviral effect, albeit weak, against the Newcastle virus (NDV), which causes serious diseases in poultry and thereby substantial financial loss (Elizondo-Gonzalez et al., 2012).

The first seaweed compound with antiviral activity against Canine Distemper Virus (CDV), a morbillivirus related to the measles virus that infects dogs and other carnivores (Trejo-Avila et al., 2014), was reported in 2014. In the same year, another report showed the antiviral potential of brown algae products against norovirus infections using the Feline Calicivirus as a model (Choi et al., 2014).

The Griffithsin protein (GRFT protein), produced by a red alga called *Griffithsin* sp., has activity against viruses such as the human immunodeficiency virus (HIV), hepatitis C virus (HCV), human papillomavirus, herpes simplex virus 2 (HSV-2) and the Japanese encephalitis virus (JEV), the most frequent cause of viral encephalitis in Asia (Ishag et al., 2013; Takebe et al., 2013). GRFT also shows antiviral activity against respiratory viruses including SARS-CoV (Urbani strain), coronaviruses (HCoV-NL63 group and HCoV-OC43 group) and infectious bronchitis virus (IBV; Mori et al., 2005; O’Keefe et al., 2009, 2010; Meuleman et al., 2011; Ishag et al., 2013; Takebe et al., 2013; Levendosky et al., 2015; Lusvarghi and Bewley, 2016). Studies with SARS-COV suggest that the GRFT also can reduce the overall viral load, and it acts by binding to the peak glycoprotein at the start of an infection and as an immunomodulator (O’Keefe et al., 2009, 2010). The GRFT protein of red algae is also effective against the porcine diarrhoea virus (PEDV), which causes deaths and large economic losses in the swine industry (Li et al., 2019).

Human noroviruses cause gastroenteritis and phlorotannins from macroalgae have been reported to be active against murine norovirus (MNV), a model for human noroviruses (Eom et al., 2015).

The red seaweed compound carrageenan prevents the replication of Rhinoviruses (HRV1A, HRV2, HRV8, HRV14, HRV16, HRV83, and HRV84), which are more common causes of infection than any other respiratory virus. Carrageenan has also been reported to help improve symptoms of common cold and reduce high viral loads (Grassauer et al., 2008; Eccles et al., 2010). The meroditerpene epitaondiol extracted from brown macroalgae has virucidal activity the human metapneumovirus (HMPV), another respiratory virus, and inhibits the penetration of viral particles into cells (Mendes et al., 2010).

Fucoidan and another polysaccharide from a brown seaweed have virucide activity against Influenza A (H5N1), Hantavirus and Respiratory syncytial virus (RSV), and also other non-respiratory viruses (Deryabin et al., 2014; Cao et al., 2016; Pavliga et al., 2016).

A sulfated polysaccharide was isolated from the microalgae *Spirulina platensis* and called calcium spirulana (Ca-SP), this isolate showed antiviral activity against replication of several enveloped viruses, including Herpes simplex virus type 1, human cytomegalovirus, measles virus, mumps virus, influenza A virus, and HIV-1 (Hayashi et al., 1996).

### Viral Action Mechanism From Bioactive Algae

Viruses are obligatorily intracellular parasites; they need to invade cells and hijack cellular machinery to replicate. Enveloped viruses tend to fuse their membrane to the cell membrane to release the genome into the cytoplasm of the host using cytoplasmic endosomes. Fusogenic peptides active at low pH facilitate access to cytoplasmic endosomes; thus, preventing pH lowering by molecules released by cells inhibits virion fusion. Non-enveloped viruses, such as enteroviruses, accumulate in endosomes which are highly acidic. Recognition depends on the activity of receptors on the surface of the cells, where the virus binds. Most enteroviruses bind α2β1 integrin and adenoviruses and coxsackieviruses use adenovirus and coxsackie receptors (Belgerson et al., 1997; Marjomäki et al., 2015). Viruses with an RNA genome initiate their translation and transcription in the cytoplasm, such that they are specific potential targets for viral inhibitors inside the cell. DNA viruses need to penetrate the nucleus to start the process of replication. During translation and transcription, there is an abundance of proteins and viral polymerases, which are also potential targets for inhibition. Non-enveloped viruses are assembled in the cytoplasm in general, and this is followed by cell lysis and thus release of infectious viral particles (Linnakoski et al., 2018).

The antiviral mechanism of compounds obtained from algae is generally related to their specific structure and type of virus. Thus, each algae biomolecule may have a distinct mechanism to inactivate different types of viruses. Some studies suggest that one of the mechanisms of action involved in viral inactivation by algae is due to algal cells having a negatively charged surface which, interacting with the positive charge present in viruses or their cell surfaces, can prevent entry and cellular virus replication (Buck et al., 2006; Grassauer et al., 2008; Branyikova et al., 2018; Eccles, 2020). Furthermore, this antiviral mechanism that algae present is due to the synergistic effect that can occur with the combined use of different compounds present in these algae (Rosales-Mendoza et al., 2020). Some mechanisms of action of distinct and several compounds from algae with antiviral potential are described below.

Sulphated polysaccharides from seaweed have antiviral effects by acting at the beginning of the virus infection, interfering with virus adsorption and internalization (Mazumder et al., 2002; Talarico et al., 2004; Matsuhiro et al., 2005; Wang et al., 2012).

The antiviral action of fucoidan consists of blocking viral adsorption, inhibiting viral penetration and replication, and heavily suppressing virus-induced syncytium (Ponce et al., 2003; Mandal et al., 2007; Trejo-Avila et al., 2014).

Iota-carrageenan from red marine algae acts against infection by influenza virus through direct binding of the polymer to the viral particles, thereby preventing adsorption onto cell receptors and subsequent internalization, iota-carrageenan has a long chain of negatively charged molecules that attract and capture positively charged viruses and prevent them infecting cells (Leibbrandt et al., 2010; Eccles, 2020). Iota-carrageenan also can bind to the surface of the rhinovirus and causes inhibition of the virus’s binding to cell receptors (Grassauer et al., 2008). Iota-carrageenan has an inhibitory effect also after the virus enters the cell, blocking mandatory conformational changes of rhinovirus, Iota-carrageenan acts on the occlusion of the virion surfaces involved in binding to the cellular proteins involved in the infectious process, which can prevent replication and also generate defective viral particles (Buck et al., 2006; Grassauer et al., 2008).

Carrageenan from red seaweed adsorbs Enterovirus 71 particles, consequently preventing the viruses from entering cells (Chiu et al., 2012). Both λ- and ι-carrageenans can effectively interfere with the adsorption and internalization of DENV when added at the same time as the virus or shortly after infection.

Griffithsin protein from the macroalga *Griffithsia* sp. has the ability to bind to specific oligosaccharides in the glycoproteins in the virus envelope and block viral entry, GRFT was active against SARS-CoV and HCoV-NL63 using protein-protein interactions for viral targeting, and for HCoV-OC43 and IBV-CoV utilize protein-carbohydrate interactions for viral attachment (O’Keefe et al., 2010).

An extract from the brown algae *Laminaria japonica* has a polysaccharide that efficiently inhibits RSV replication, and the mechanism of action depends on interferon regulatory factor 3 (IRF-3)-mediated interferon-alfa (IFN-α) secretion (Cao et al., 2016).

The antiviral effects of macroalgae extracts against the HMPV virus involve interaction with the viral particles outside the cells and thereby preventing infection. Meroditerpenoids of this species have both virucidal effects and the capacity to inhibit the penetration of viral particles into cells. The binding of HMPV to heparan sulfate involves charge-charge, heparan sulfate blocks the binding of HMPV to the receptor and this occlusion inhibits infection of cells (Klimyte et al., 2016).

**FIGURE 1:**
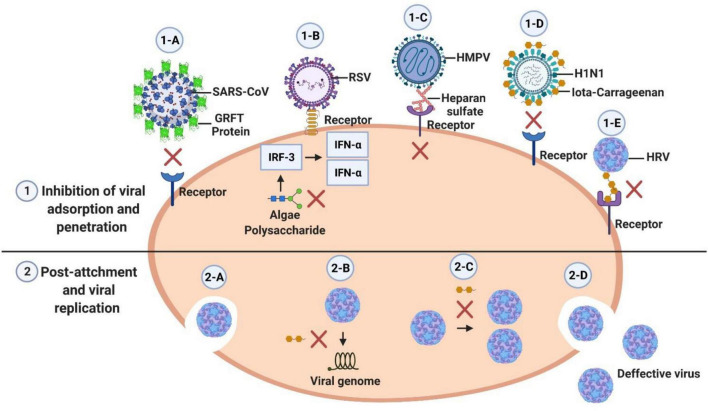
The mechanisms of the action of natural compounds can be divided into two phases: before and after viral entry. 1-A: GRFT Protein from the *Griffithsia* sp. macroalgae binds to specific oligosaccharides in the virus envelope glycoproteins and block viral entry (O’Keefe et al., 2010). 1-B: Polysaccharides from *Laminaria japonica* enhance the expression level of IRF3 and the secretion of IFN alpha that results an antiviral activity against RSV. 1-C: The binding of HMPV to heparan sulfate involves charge-charge interactions; this blocks the binding of HMPV to the receptor and consequently inhibits the infection of cells (Klimyte et al., 2016) 0.1-D: Iota-carrageenan from red algae has a long chain of negatively charged molecules that attract and capture positively charged viruses and prevent them from infecting cells (Leibbrandt et al., 2010; Eccles, 2020). 1-E: Iota-carrageenan binds to the surface of rhinovirus and inhibits virus binding to cell receptors (Grassauer et al., 2008). 2-A: Viral entry. 2-B Iota-carrageenan also has an inhibitory effect after the virus enters the cell, blocking the conformational changes of rhinovirus necessary for infection (2-B: uncoating and 2-C: replication) Iota-carrageenan acts occludes virion surfaces involved in binding to cellular proteins required for the infectious process; this prevents replication and results in the viral particles produced being defective (Buck et al., 2006; Grassauer et al., 2008). 2-D: Exit of defective viral particles.

Many groups who have demonstrated that compounds and extracts from macroalgae have antiviral effects report the need for studies to elucidate the mechanisms of action of the bioactive compounds (Choi et al., 2014; Eom et al., 2015). Figure 1 illustrates various mechanisms of antiviral activity of compounds derived from algae.

### Antiviral Drugs From Algae as Alternative to Synthetic Drugs

Algae are a natural source of compounds with antiviral properties, have proven efficiency against enveloped and non-enveloped viruses, this compounds and extracts from algae have inexpensive to obtain, especially those of marine origin (Alam et al., 2021). Furthermore, algae are an alternative resource to synthetic drugs, because algae have very low toxicity and some are non-toxic at doses that have a broad antiviral spectrum against several viruses and minimal side effects (Besednova et al., 2021). Among the benefits of algae, we can still mention that due to the diversity of molecules and their mechanisms of action, it inactivates viruses and block their action without causing resistance or selection of these organisms (Hamed et al., 2015).

## Conclusion

Various compounds from algae have potent activities against viruses, and are strong candidates for the control and treatment of viruses that affect humans and animals. These bioactive compounds should be further explored for health applications, both in clinicals and the environmental. They are also promising for use as low toxicity sanitizers of high virucidal capacity. More studies are required both for prospecting algae for active molecules and for the development of products suitable for applications in viral control.

## Author Contributions

JR, RC, AC, IT, DR-L, and GF: original draft preparation and manuscript reviewing and editing. GF: final manuscript supervision. All authors contributed to the article and approved the submitted version.

## Conflict of Interest

The authors declare that the research was conducted in the absence of any commercial or financial relationships that could be construed as a potential conflict of interest.

## Publisher’s Note

All claims expressed in this article are solely those of the authors and do not necessarily represent those of their affiliated organizations, or those of the publisher, the editors and the reviewers. Any product that may be evaluated in this article, or claim that may be made by its manufacturer, is not guaranteed or endorsed by the publisher.
